# Modeling habitat suitability for the lesser‐known populations of endangered mountain nyala (*Tragelaphus buxtoni*) in the Arsi and Ahmar Mountains, Ethiopia

**DOI:** 10.1002/ece3.11235

**Published:** 2024-04-15

**Authors:** Ejigu Alemayehu Worku, Paul H. Evangelista, Anagaw Atickem, Afework Bekele, Jakob Bro‐Jørgensen, Nils Chr. Stenseth

**Affiliations:** ^1^ Centre for Ecological and Evolutionary Synthesis (CEES), Department of Biosciences University of Oslo Oslo Norway; ^2^ Natural Resource Ecology Laboratory Colorado State University Fort Collins Colorado USA; ^3^ Department of Zoological Sciences Addis Ababa University Addis Ababa Ethiopia; ^4^ Mammalian Behaviour and Evolution Group, Department of Evolution, Ecology and Behaviour University of Liverpool Neston UK

**Keywords:** Boosted Regression Tree, habitat suitability modeling, Maxent, mountain nyala, *Tragelaphus buxtoni*

## Abstract

Habitat suitability models have become a valuable tool for wildlife conservation and management, and are frequently used to better understand the range and habitat requirements of rare and endangered species. In this study, we employed two habitat suitability modeling techniques, namely Boosted Regression Tree (BRT) and Maximum Entropy (Maxent) models, to identify potential suitable habitats for the endangered mountain nyala (*Tragelaphus buxtoni*) and environmental factors affecting its distribution in the Arsi and Ahmar Mountains of Ethiopia. Presence points, used to develop our habitat suitability models, were recorded from fecal pellet counts (*n* = 130) encountered along 196 randomly established transects in 2015 and 2016. Predictor variables used in our models included major landcover types, Normalized Difference Vegetation Index (NDVI), greenness and wetness tasseled cap vegetation indices, elevation, and slope. Area Under the Curve model evaluations for BRT and Maxent were 0.96 and 0.95, respectively, demonstrating high performance. Both models were then ensembled into a single binary output highlighting an area of agreement. Our results suggest that 1864 km^2^ (9.1%) of the 20,567 km^2^ study area is suitable habitat for the mountain nyala with land cover types, elevation, NDVI, and slope of the terrain being the most important variables for both models. Our results highlight the extent to which habitat loss and fragmentation have disconnected mountain nyala subpopulations. Our models demonstrate the importance of further protecting suitable habitats for mountain nyala to ensure the species' conservation.

## Graphical abstract

Habitat suitability models have been used to determine the potential suitable habitat and the environmental factors that affect the distribution of mountain nyala (*Tragelaphus buxtoni*) in the Arsi and Ahmar Mountains, Ethiopia.

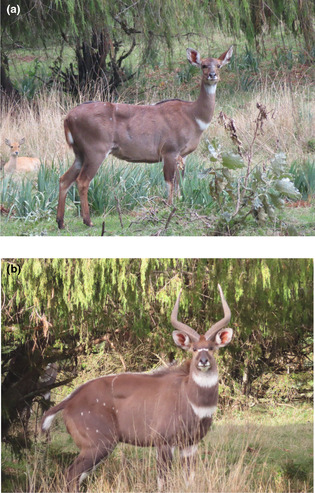

## 1 INTRODUCTION

The mountain nyala (*Tragelaphus buxtoni*) is an endemic antelope species to the southern Ethiopian Highlands where it is found in the Bale, Arsi, and Ahmar Mountains (Brown, 1969; Evangelista et al., 2012). The total population was estimated to be 7000–8000 in the 1980s (Hillman, 1986), but more recent estimates suggest populations have been reduced to approximately 4000 individuals (Atickem et al., 2011). The International Union for the Conservation of Nature (IUCN) Red List classifies the species as *Endangered* due to its small population size and restricted range, estimating the total number of mature individuals to be between 1500 and 2500 individuals (IUCN, 2016). The largest mountain nyala population occurs in the Bale Mountains (Evangelista et al., 2008), where population estimates vary between 1500–2000 (Sillero‐Zubiri, 2013) and 3756 individuals (Atickem et al., 2011). In the Arsi and Ahmar Mountains, Malcolm and Evangelista (2011) estimated mountain nyala populations totaled 980 individuals in the early 2000s across fragmented subpopulations, while EAW (unpublished data) estimated population numbers at 736 individuals in 2016. Although population numbers are low in both the Arsi and Ahmar Mountains, they are of high conservation value, providing important contributions to the species' genetic diversity and constituting a back‐up population in the event the Bale Mountain population declines further.

**FIGURE 1:**
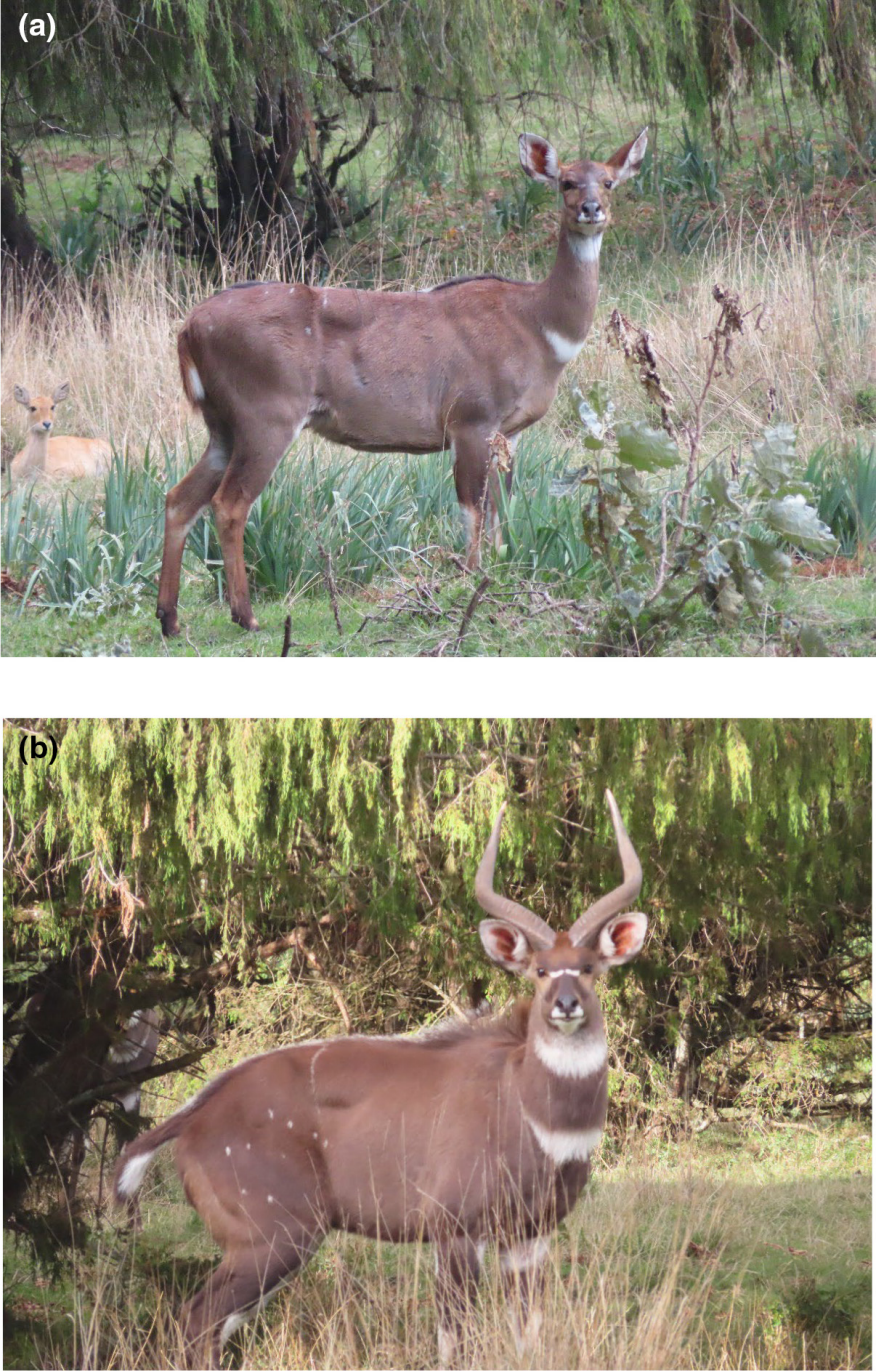
Mountain nyala (*Tragelaphus buxtoni*) (a, female; b, male).

Mountain nyala (Figure 1) are generally restricted to elevations ranging from 1600 m to 4300 m a.s.l., where their natural habitats include montane forests, *Erica* heathlands, and the Afro‐alpine vegetation zones (Evangelista et al., 2007). However, mountain nyala will also utilize plantation forests and other landcover types that provide thermal cover and refuge from people and predators (Evangelista et al., 2007). The mountain nyala population in the Arsi and Ahmar Mountains have been displaced from much of their previous ranges, primarily due to the loss of montane forest, intensive clearing of heathlands, encroachment of settlements and livestock, and expansion of high‐altitude cultivation (Atickem et al., 2011; IUCN, 2016; Stephens et al., 2001). The conversion of diverse ecosystems into agricultural farms is reducing the landscape's heterogeneity, degrading, and fragmenting what little habitat remains. Livestock overgrazing is also a significant environmental issue in these mountains, as it can have negative impacts on both the habitat and wildlife conservation efforts (Stephens et al., 2001). Livestock overgrazing diminishes the availability of habitat for wildlife and results in forest cover loss that affects both the ecosystem and the communities depending on these forests for their livelihoods (Mekuria & Aynekulu, 2013). As a result, wildlife managers are prioritizing strategies to protect critical habitat, such as the designation of Arsi Mountains National Park established in 2011.

The full range of mountain nyala was first mapped by using coarse‐scale climatic, topographic, and vegetation variables by Evangelista et al. (2007, 2008), however, the species' historical range was likely much greater. Higher resolution habitat suitability maps were later developed for the Bale Mountains, relying heavily on remotely sensed vegetation indices and dominant land cover types (Atickem et al., 2011; Evangelista et al., 2012). These studies estimated suitable habitat to cover from 2857 km^*2*^ (Evangelista et al., 2012) to 8333 km^2^ (Atickem et al., 2011). However, we found no recent studies on the potential suitable habitat and the environmental factors limiting the distribution of mountain nyala populations in the Arsi and Ahmar Mountains.

Geospatial technologies, such as geographic information systems (GIS) and remote sensing, combined with various geospatial modeling techniques, allow researchers to model habitat suitability and help assess the importance of environmental conditions for a single species (Evangelista et al., 2017). Habitat suitability models relate species' occurrence with associated environmental features, such as climate and topography (Austin, 2007; Elith & Leathwick, 2009; Evangelista et al., 2017; Guisan & Zimmermann, 2000; Li & Wang, 2013). Many modeling techniques are available to explore the relationship between the occurrence of a species and predictor variables (Franklin, 2010). The most widely used methods of habitat suitability modeling include Boosted Regression Trees (BRT), Maximum Entropy (Maxent), and random forest (García‐Callejas & Araújo, 2016). Maxent is one of the most widely used models to estimate the potential distribution of wildlife species (Phillips et al., 2006), including the computation of variable response curves. It can produce accurate results with a large number of randomly generated pseudo‐absence points (Phillips & Dudík, 2008) and is less sensitive to small sample sizes. Because the various algorithms used to model species distributions sometimes produce varying results depending on the type and quantity of data inputs (Evangelista et al., 2017; Franklin, 2010; Guo et al., 2015), it can be desirable to combine the different model algorithms to create an ensemble model, which provides a more robust result than if relying on a single model only (Forester et al., 2013; Stohlgren et al., 2010).

In this study, we intended to increase our understanding of mountain nyala and habitat requirements for populations in the Arsi and Ahmar Mountains in order to support long‐term conservation and management priorities. These priorities include the identification of natural habitats and their protection from human disturbances such as intensified agriculture expansion, livestock grazing, habitat loss, and degradation, which can be achieved by strategies including the establishment of protected areas and land use land cover monitoring.

We used two different habitat suitability models proven to be effective for predicting distributions of endangered wildlife; BRT (Elith et al., 2008) and Maxent (Phillips et al., 2006). Various remotely sensed data products have been widely used to model habitat suitability for different species (Feilhauer et al., 2012). One of the most common variables derived from remote sensing is land cover type, which influences species ranges (Blach‐Overgaard et al., 2010; Franklin, 2010). Topographic variables like elevation and slope have been used to model habitat suitability and used as proxies for micro‐climate conditions (Atickem et al., 2011; Moradi et al., 2019). For example, elevation has a considerable effect on the temperature regime and precipitation, while the steepness of the slope affects soil moisture and soil properties (Franklin, 2010). In addition to these predictor variables, the most commonly remotely sensed indices in wildlife modeling is the Normalized Difference Vegetation Index (NDVI), calculated as (NIR‐Red)/(NIR + Red), where NIR and Red are the amount of near‐infrared and red reflectance of healthy green vegetation (Pettorelli, 2013). NDVI can be considered as a proxy for habitat quality (Muposhi et al., 2016) and by providing information on vegetation distribution and health, it constitutes a helpful tool for examining the ecology of large herbivore species (Pettorelli et al., 2011). Additionally, the tasseled cap vegetation indices have also been used in the habitat suitability modeling of endangered species (Treglia et al., 2015). The tasseled cap indices include the green vegetation index (greenness), soil brightness index (brightness), and moisture index (wetness), indicating moisture retained by vegetation and soil (Campos et al., 2016; Parviainen et al., 2013; Zielinski et al., 2015).

Our main research objectives are to: (1) model suitable habitat for mountain nyala using occurrence data collected from the field, (2) identify key environmental conditions that are associated with the current distribution of the critically important mountain nyala population in the Arsi and Ahmar Mountains specifically, and (3) provide wildlife managers with important information to develop and implement meaningful long‐term conservation strategies for this endangered species.

## 2 METHODS

### 2.1 Study area

**FIGURE 2:**
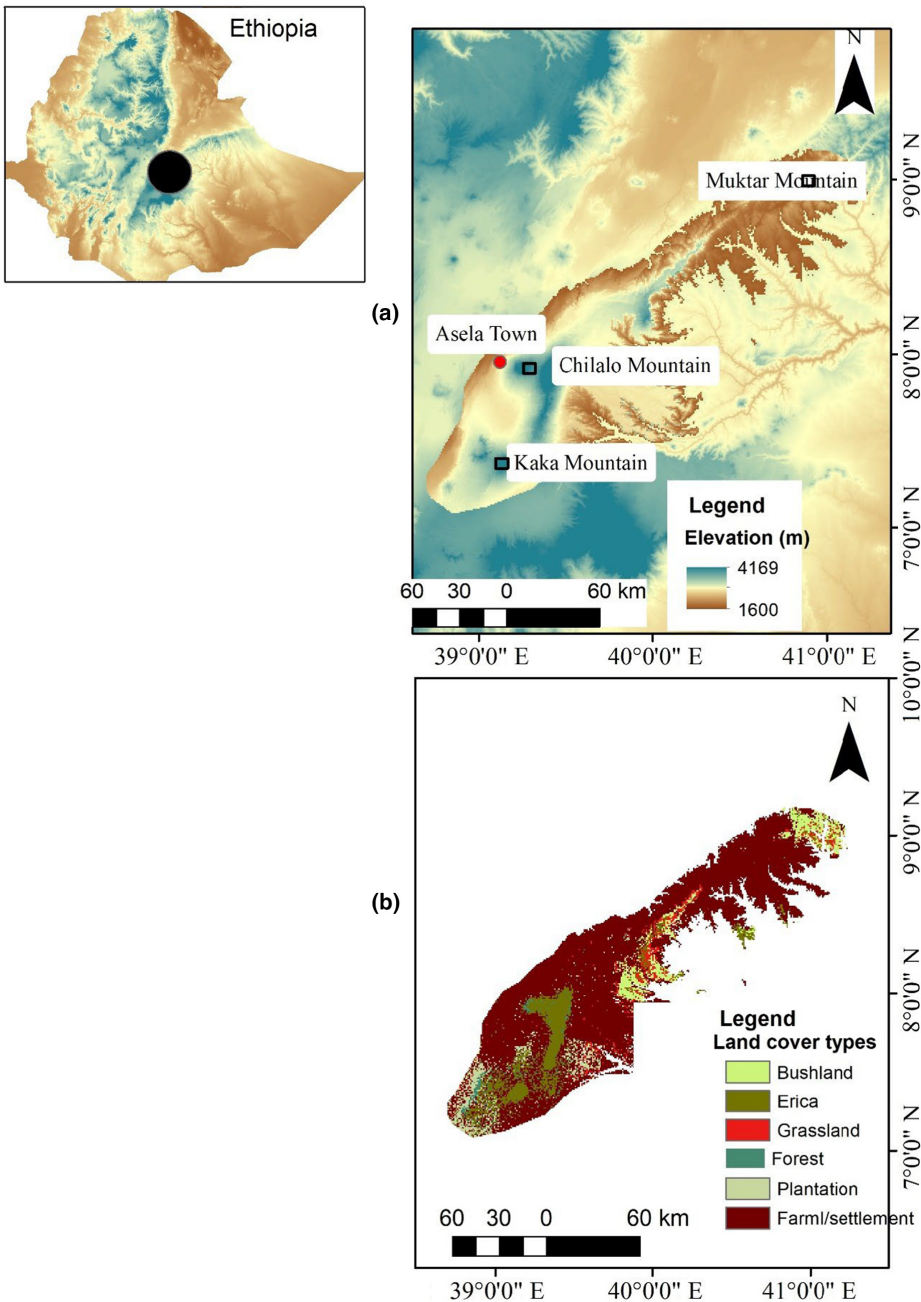
Elevation (a) and land cover types (b) map of the Arsi and Ahmar Mountains, Ethiopia.

Our study was conducted in the Arsi and Ahmar Mountains which lie east of the Rift Valley in south‐central Ethiopia (Figure 2). The study area is ca. 20,567 km^2^ with elevations ranging from 1600 to 4169 m a.s.l. Annual rainfall occurs in a bimodal pattern, with the main rainy season occurring between June and September. The dry season lasts from November to February, followed by the short rainy season during March and April. The Worldclim database (1979–2013) reports that the annual mean temperature ranges from 3 to 24°C, and the annual mean rainfall ranges from 657 to 1652 mm (Fick & Hijmans, 2017).

The study areas were historically covered by montane forests (1600–2300 m), an ericaceous belt (2300–3200 m), and Afro‐alpine vegetation (>3700 m) (Bekele‐Tesemma et al., 1993; Uhlig, 1988). The high variability in ecological conditions, topography, and edaphic conditions contribute to the vast diversity of flora and fauna in the region (Brown, 1969; Girma et al., 2018; Hedberg, 1971). Most of the montane forests have been cleared for settlements, agriculture, and livestock grazing. However, remnants of these forests can still be found on steep slopes of both mountain ranges. These forests are dominated by *Hagenia abyssinica*, *Dombeya torrida*, *Discopodium penninevum*, *Schefflera abyssinica*, *Juniperus procera*, and *Hypericum revolutum* (Evangelista et al., 2007; Hedberg, 1951). There are scattered forestry plantations throughout the study area, but primarily concentrated in the southern part of the Arsi Mountains. These areas have been reforested with *Hagenia abyssinica*, *Eucalyptus globulus*, *Cupressus lusitanica*, *Pinus patul*a, and *Pinus radiata*. The vegetation types of the ericaceous belt are characterized by shrubs and shrubby trees, including *Hypericum revolutum*, *Erica trimera*, and *Erica arborea* (Friis et al., 2010; Miehe & Miehe, 2000). The Afro‐alpine vegetation provides a habitat for several threatened endemic species, including the endangered Ethiopian wolf (*Canis simensis*) (Ashenafi et al., 2005; Marino, 2003; Sillero‐Zubiri et al., 2011) and endemic Menelik's bushbuck (*Tragelaphus scriptus meneliki*) (Girma et al., 2015).

### 2.2 Species occurrence data collection

We assessed the presence of mountain nyala using fecal pellet counts in plots placed along transects throughout the study area in 2015 and 2016 (Table S1). Throughout the survey, we differentiated mountain nyala pellets from those of other sympatric herbivores, such as Menelik's bushbuck, based on their size and shape. The mountain nyala pellets are cone‐shaped with a pointed end and have a relatively larger diameter, whereas the pellets of Menelik's bushbuck are smaller and elongated (Girma et al., 2015). A total of 196 transects were established randomly, and 1710 plots were sampled and surveyed for the presence of mountain nyala fecal pellets. A minimum distance of 1 km was set between transects to avoid an overlap of sampling points. The location of fecal pellets was recorded using a Global Positioning System (GPS) and was designated as a presence point. Presence points were binned into 30 m × 30 m cells, retaining only one presence point per grid cell to avoid biases and to match the environmental data resolution (Evangelista et al., 2008).

### 2.3 Predictor variables

We used seven predictor variables derived from multiple remote sensing sources to build the habitat suitability models (Table 1). All image preprocessing, such as mosaic and layer stacking, were conducted using ArcGIS v. 10.4.1 (ESRI, www.esri.com). We used NASA's Shuttle Radar Topography Mission (SRTM) digital elevation dataset (http://srtm.csi.cgiar.org) for elevation and to derive slope (Sappington et al., 2007). Cloud‐free scenes from Landsat 8 Operational Land Imager (OLI) were downloaded from the US Geological Survey Earth Resources Observation and Science (EROS) Center (https://earthexplorer.usgs.gov/) for classifying land cover types. Images were classified based on supervised image classification using 420 ground‐truthing points with a maximum likelihood classifier algorithm (Fetene et al., 2016). Ground control points for the image classification were collected using a handheld GPS during field observations in 2015 and 2016. We also assessed accuracy using an additional 420 reference points that were not used in the signature development. The results showed a strong overall classification performance, with an accuracy of 85.4%. Land cover was classified into six types: montane forest, *Erica* shrub, bushland, grassland, plantation forest, and agriculture/settlement (Table 1) and included as a categorical variable in the analysis.

**TABLE 1:** Predictor variables used for modeling habitat suitability for mountain nyala (*Tragelaphus buxtoni*) used in the study.

Predictor variable	Description
Land cover types	
*Erica*	Dominated by *Erica trimera* and *E. arborea* shrubs, usually more than 0.5 m and <5 m in height
Montane forest	Dominated by native trees, including *Juniperus Procera*, *Olea* spp., and *Podocarpus falcatus*
Grassland	Dominated by native grasses and herbs (Kindu et al., 2013)
Farmland and settlements	Dominated by agricultural lands, homesteads, and urbanized areas (Kindu et al., 2013)
Bushland	Dominated by woody shrubs of mixed species, with low stature between 3 and 7 m in height (Fetene et al., 2016)
Plantation	Dominated by plantation forests of *Pinus patula*, *P. radiata*, *Cupressus lusitanica*, and *Eucalyptus* spp.
Topography	
Elevation (meters)	Height above sea level derived from NASA's Shuttle Radar Topography Mission (SRTM) digital elevation dataset (http://srtm.csi.cgiar.org)
Slope (degrees)	The steepness of a surface/angle of incline
Vegetation productivity	
Normalized Difference Vegetation Index (NDVI)	Non‐linear transformation of the ratio between band 3 (red) and band 4 (near‐infrared) of Landsat and used as an index of vegetation biomass, density and canopy cover (Pettorelli et al., 2005)
Tasseled cap indices	
Greenness	A measure of photosynthetically active vegetation (Kauth & Thomas, 1976)
Wetness	A measure of soil and vegetation moisture content (Kauth & Thomas, 1976)
Brightness	A measure of bare soil (Kauth & Thomas, 1976)

We also derived NDVI and tasseled cap indices representing brightness, greenness, and wetness from the Landsat images (Baig et al., 2014). All raster images were clipped and resampled to the same extent and resolution (30 m x 30 m resolution).

To avoid overfitting and minimize multicollinearity, we used Pearson's rank correlation, and pairs with high correlation values (>0.70) were identified and excluded from the modeling process (Dormann et al., 2013) (Table S2). Six predictor variables were used to model habitat suitability for mountain nyala, and brightness was excluded from further analysis due to its strong intercorrelation with the other variables. The selected predictors include land cover types, NDVI, elevation, slope, greenness, and wetness.

### 2.4 Habitat suitability modeling

To model the suitable habitat of mountain nyala, we used two machine learning methods: (1) BRT, based on boosting techniques (Elith et al., 2006); and (2) Maxent, based on parametric maximum likelihood (Phillips et al., 2006). All the models were built in R (R Development Core Team, 2021) version 4.0.5. A total of 130 presence points were used for each model. We ran 10‐fold cross‐validation in which 90% of the data were chosen for model training and tested each model by withholding 10% of the data (Guisan et al., 2017).

For the BRT approach, we built models by using different combinations of learning rate (values ranging from 0.001 to 0.05) and tree complexity (1–5) from the “gbm.step” function in the gbm package (Greenwell et al., 2019) and used to compute the relative contribution of each predictor variable (Elith et al., 2008). We generated pseudo‐absence points at random that were equal in number to the presence points, following the recommendations of Barbet‐Massin et al. (2012). Based on 10 10‐fold cross‐validation results of the model, we selected 3 for tree complexity, 0.05 for learning rate, and 0.75 for bag fraction to achieve more than 1000 trees (Elith et al., 2008).

For the Maxent models, we generated 10,000 random pseudo‐absence points throughout the study area to model the habitat suitability map. Different penalty terms related to regularization multiplier values were used to regulate model complexity and overfitting (Chala et al., 2016). We built multiple models, starting with the lowest penalty term of 0.5 and gradually increasing it until smooth response curves were found (penalty term of 3 in our case).

The probabilistic predictions in each model were split into binary presence‐absence maps using “Maximizing the sum of sensitivity and specificity” (MaxSSS) threshold (Guisan et al., 2017; Liu et al., 2005). The purpose of these binary maps was to quantify the amount of suitable (greater than MaxSSS value) and unsuitable habitat (less than MaxSSS value) for mountain nyala in the region.

An ensemble approach was used to combine model predictions (Marmion et al., 2009) to map the current critical habitat of mountain nyala. Ensemble habitat suitability models integrate predictions of several single‐algorithm models that help to reduce potential bias and uncertainty (Araújo & New, 2007; Scales et al., 2016). The ensemble approach involves combining the binary habitat maps (binary maps of suitable and unsuitable habitat) from BRT and Maxent models into a single map (Stohlgren et al., 2010). The final ensemble maps consisted of pixel values representing the number of models in agreement. In this way, the ensemble model yielded three possible pixel values; a value of zero indicates that neither model predicted suitable habitat, a value of one indicates that only one model predicted suitable habitat, and a value of two indicates that both models predicted suitable habitat.

### 2.5 Assessing the performance of the distribution models

The BRT and Maxent models were evaluated using a 10‐fold cross‐validation procedure. We used the area under the receiver operating curve (AUC) as an evaluation metric to assess the predictive performance of the models (Allouche et al., 2006). The AUC values (≥) 0.90 are being “high accuracy”; 0.70 ≤ AUC < 0.90 being “good accuracy”; 0.50 ≤ AUC < 0.70 being “low accuracy” and AUC < 0.50 being “no better than random” (Swets, 1988).

## 3 RESULTS

Our BRT and Maxent models showed high levels of predictive power, with an AUC evaluation metric of 0.96 for the final BRT model and 0.95 for the Maxent model.

### 3.1 Identifying the most suitable potential suitable habitat

**FIGURE 3:**
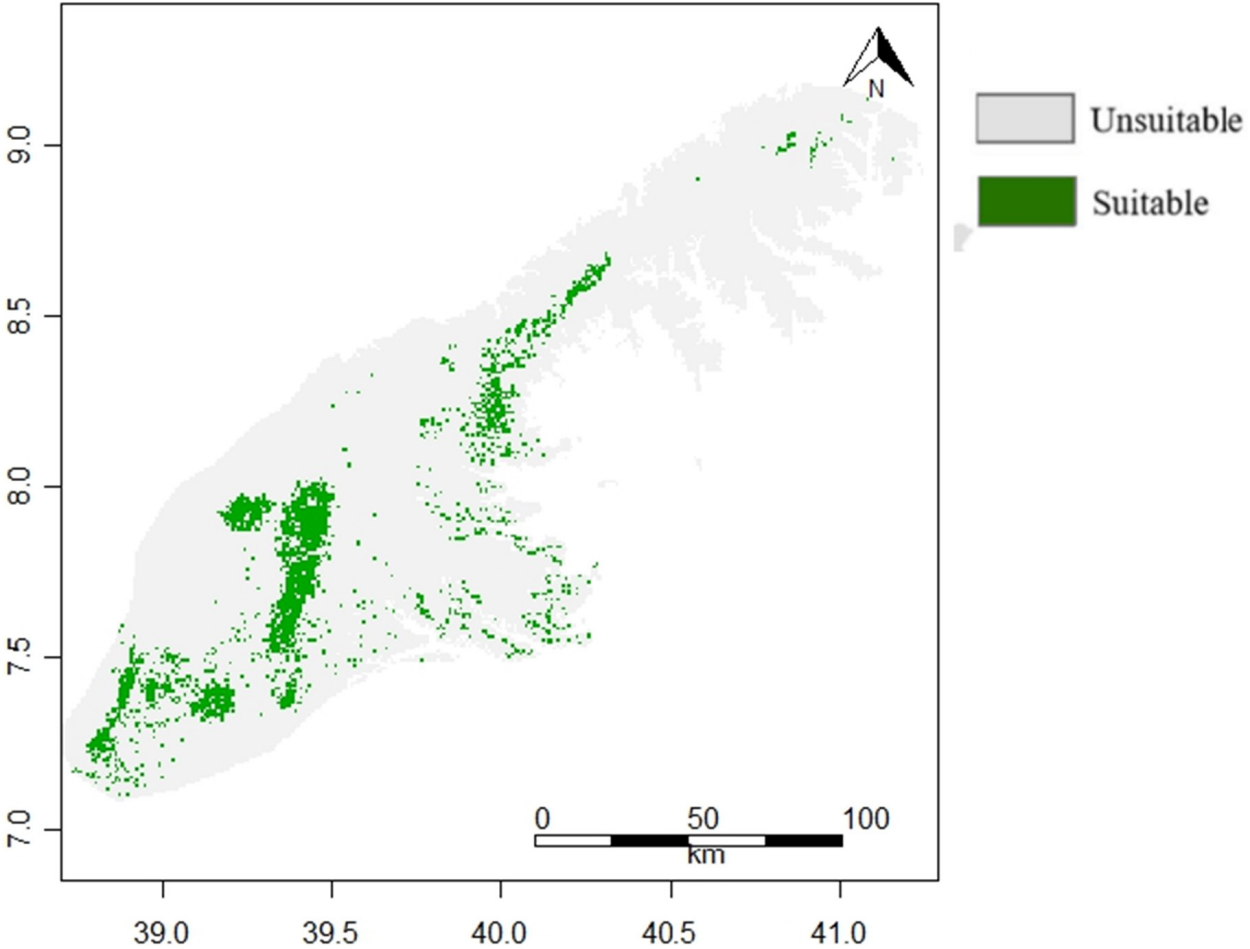
Binary output of habitat suitability model for mountain nyala (*Tragelaphus buxtoni*) in the Arsi and Ahmar Mountains, Ethiopia using the Boosted Regression Tree (BRT) model. Green dots indicate suitable areas above the absence‐presence threshold, and gray dots indicate unsuitable areas.

**FIGURE 4:**
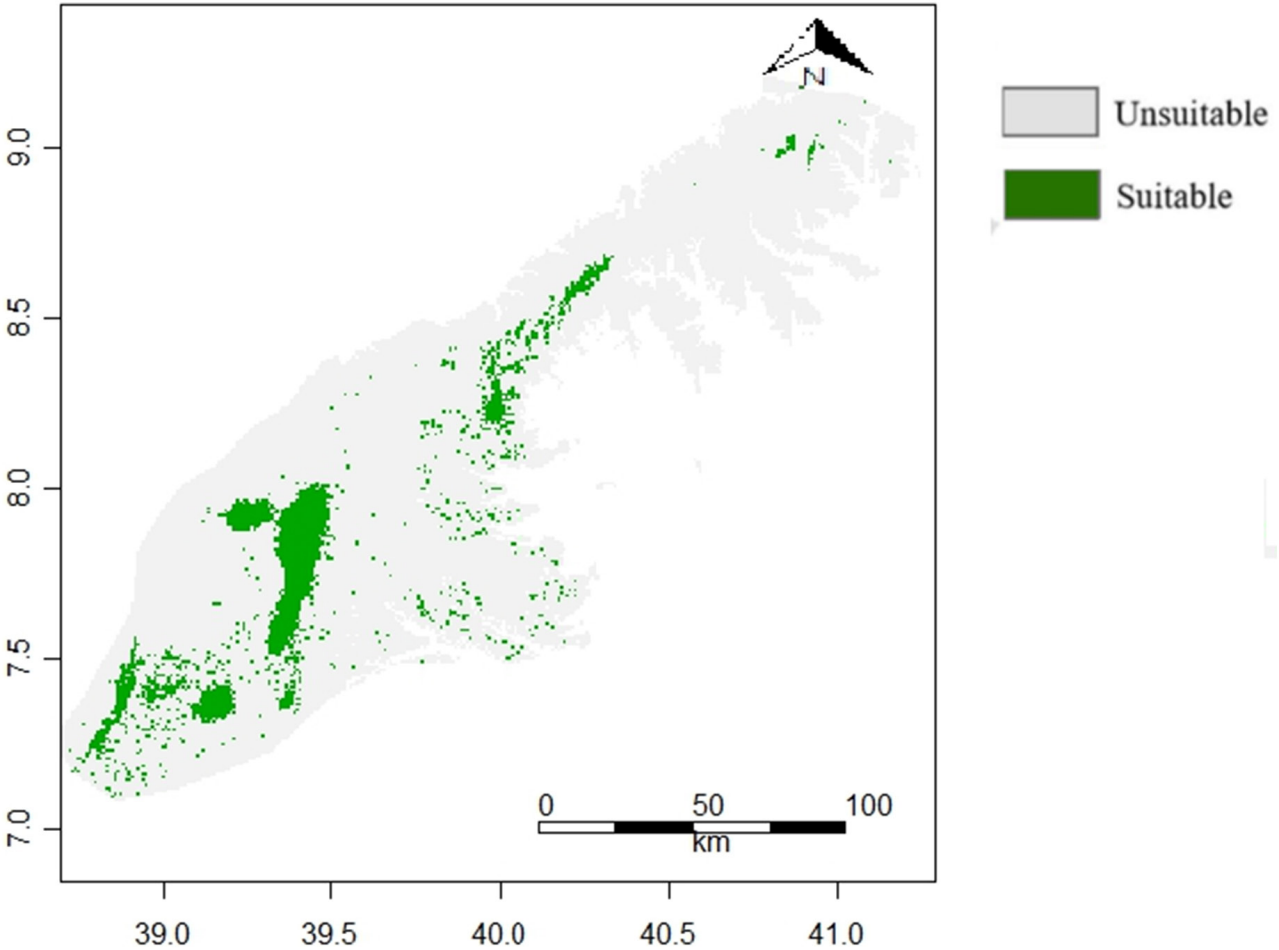
Binary output of habitat suitability model for mountain nyala (*Tragelaphus buxtoni*) in the Arsi and Ahmar Mountains, Ethiopia using the MaxEnt model. Green dots indicate suitable areas above the absence‐presence threshold, and gray dots indicate unsuitable areas.

Both models successfully recognized potentially suitable habitats. The most suitable habitat in the study area was observed in the grassland and montane forest followed by ericaceous habitat *Erica*. However, the BRT model showed a far greater habitat range than the Maxent model across the mountains. The binary classification of the habitat suitability into a suitable and unsuitable based on the threshold value also varied (Figures 3 and 4).

**FIGURE 5:**
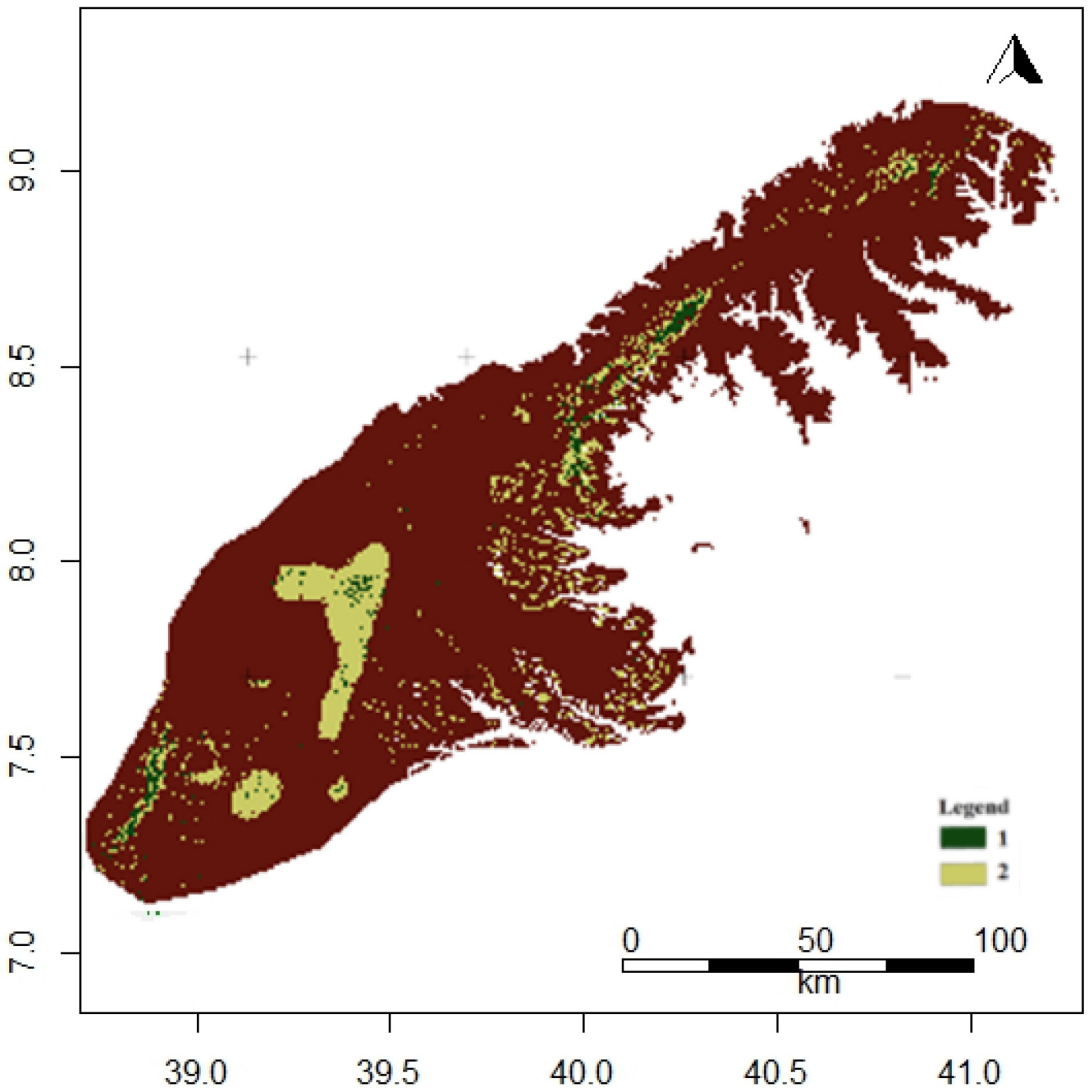
Predicted habitat suitability for mountain nyala (*Tragelaphus buxtoni*) in the Arsi and Ahmar Mountains, Ethiopia, based on an ensemble of the Boosted Regression Tree (BRT) and Maxent models. (Areas for which one model predicted suitable habitat are shown in dark green, areas for which both models predicted suitable habitat are shown in light green).

From the predictive habitat suitability maps (Figures 3 and 4) of BRT and Maxent, presence of mountain nyala was predicted to be more likely in the central and steep slope areas of the Arsi and Ahmar Mountains, and habitat suitability was higher in areas with high elevation, NDVI, and slope. Our ensemble model suggested that the potential habitat suitable for mountain nyala covered a total of 1864 km^2^ in the Arsi and Ahmar Mountains (Figure 5).

### 3.2 Environmental predictors and response curves

The main explanatory variables of habitat suitability for the mountain nyala in the BRT model were (in decreasing order of importance): land cover type (58.7%), normalized vegetation index (NDVI, 15.3%), elevation (9.9%), and slope (8.3%), resulting in a cumulative score above 92% (Table 2).

**TABLE 2:** Relative contributions of the six selected predictor variables in the BRT and Maxent models of habitat suitability for mountain nyala (*Tragelaphus buxtoni*).

Predictor variable	BRT (%)	Maxent (%)
Land cover type	58.7	37.6
NDVI	15.3	27.7
Elevation	9.9	25.3
Slope	8.3	6.0
Wetness	6.0	0.8
Greenness	3.7	2.4

**FIGURE 6:**
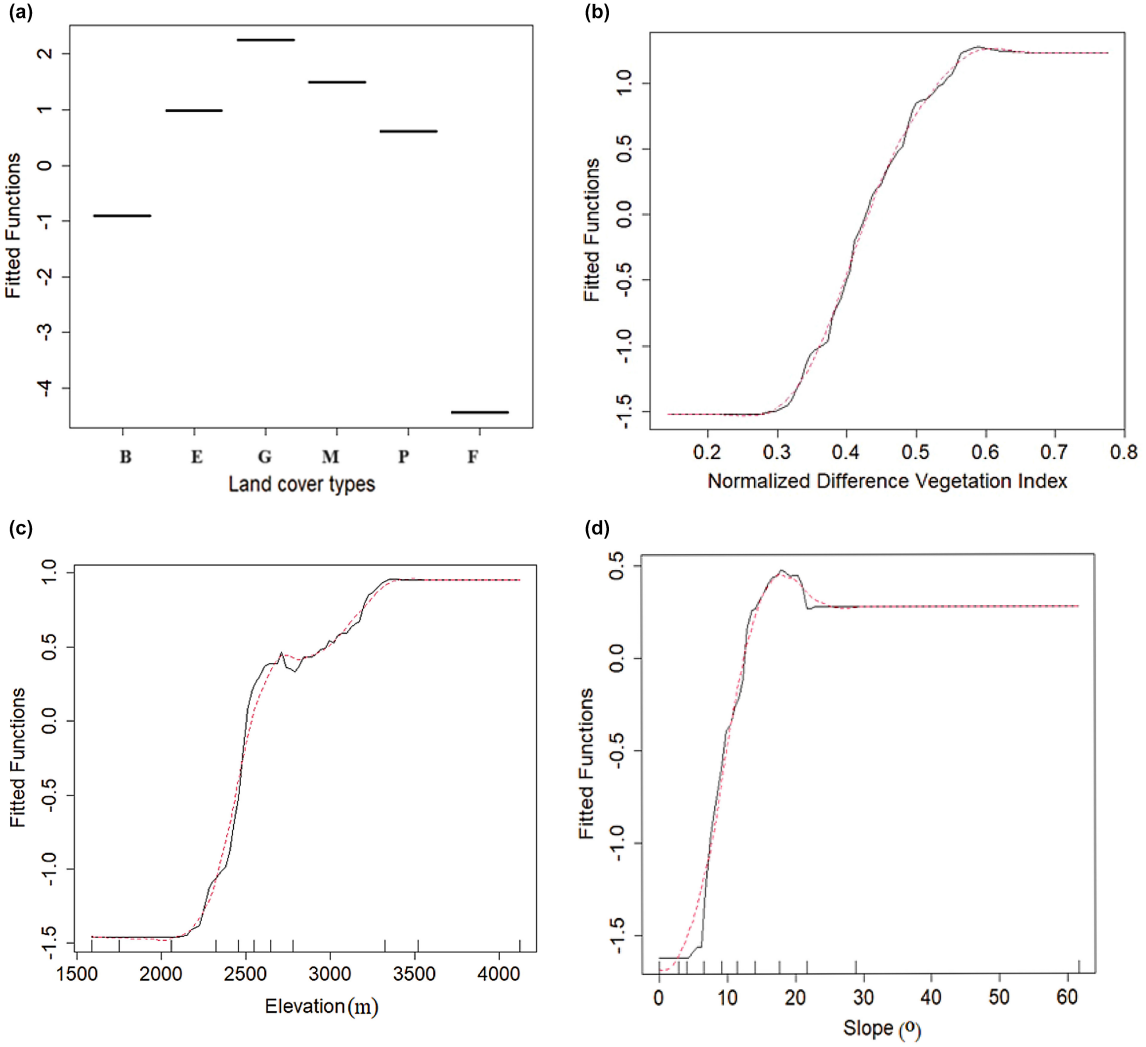
Fitted functions show the effect of the four most influential predictors on habitat suitability in the BRT model: (a) land cover type ([B] bushland, [E] Erica, [G] grassland, [M] montane forest, [P] plantation, and [F] farmland), (b) Normalized Difference Vegetation Index (NDVI), (c) elevation and (d) slope. Each of the plots shows the relationship between the respective predictor (*x*‐axis) and the response (*y*‐axis) variables while all other predictor variables were kept average.

**FIGURE 7:**
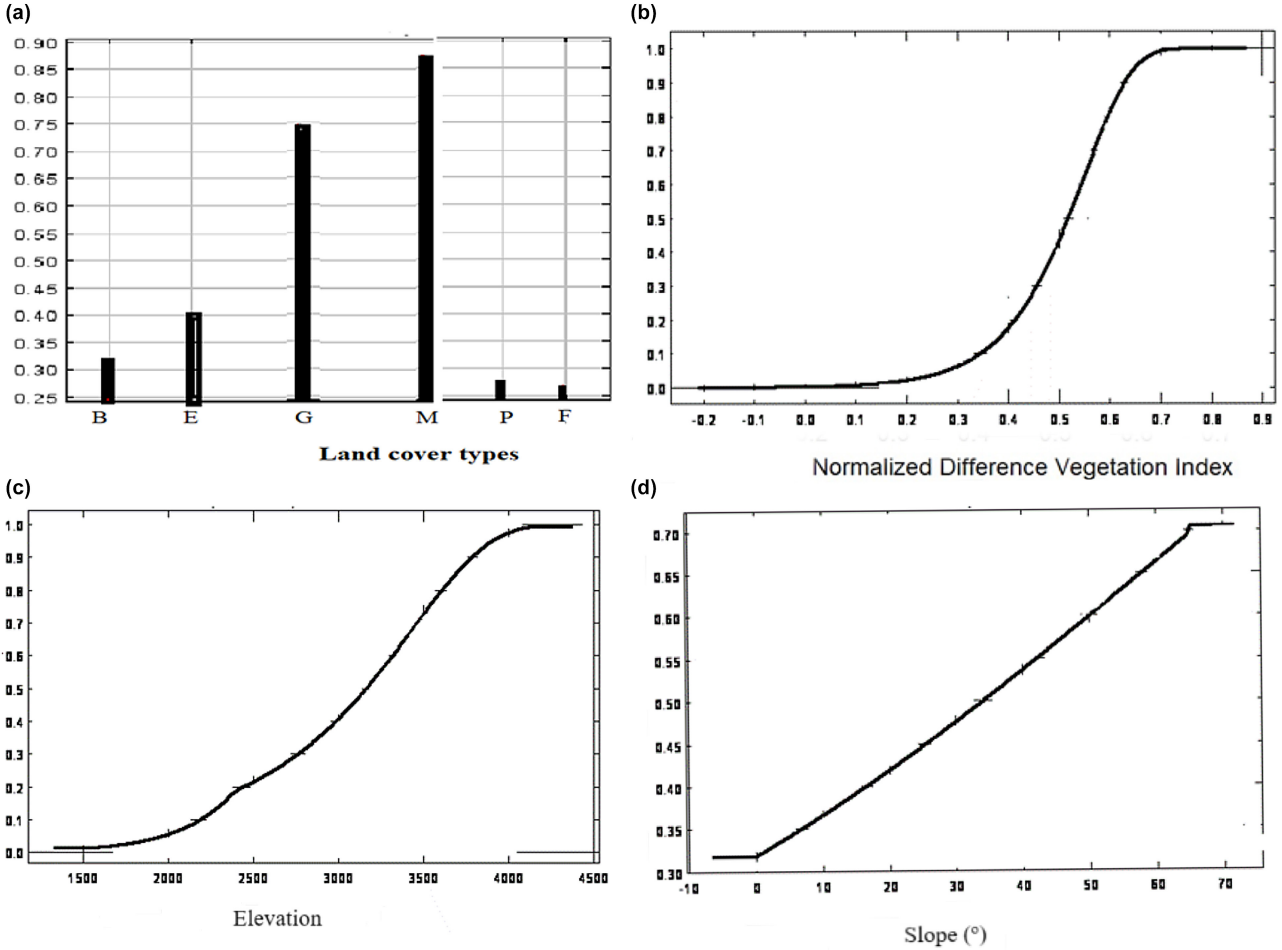
Effect of the four most influential predictors on habitat suitability of mountain nyala in Arsi and Ahmar Mountains (Ethiopia) in the MaxEnt model (a) land cover type ([B] bushland, [E] Erica, [G] grassland, [M] montane forest, [P] plantation, and [F] farmland), (b) Normalized Difference Vegetation Index (NDVI), (c) elevation and (d) slope.

The BRT response curves show the impact of the four most influential environmental variables on the predicted distribution of the mountain nyala (Figure 6). Among the land cover types, grassland and montane forest had the highest probability of mountain nyala presence, and bushland and plantations had slightly higher probability than ericaceous habitat. Non‐forest areas or farmland had very low probability of nyala presence. The response curves for NDVI, elevation, and slope showed positive correlations with habitat suitability (Figures 6 and 7).

For MaxEnt, land cover type, elevation, NDVI, and slope contributed more than 96% variable importance in the final model (Table 2), whereas wetness and greenness played a relatively modest role, contributing only <4%. Response curves varied for the four most influential variables to mountain nyala habitat suitability for MaxEnt as shown in Figure 7.

## 4 DISCUSSION

Geospatial technologies, such as geographic information systems and remote sensing combined with various modeling techniques, allow researchers to model habitat suitability and help assess the influences of environmental predictors on habitat suitability for species of conservation concern (Evangelista et al., 2017). This study estimated the potential suitable habitat and identified the ecological variables that influence habitat suitability of the endangered mountain nyala in the Arsi and Ahmar Mountains, Ethiopia.

The BRT and Maxent models showed a high degree of consistency with each other in predicting areas with suitable habitat for mountain nyala in the Arsi and Ahmar Mountains (Figures 3 and 4). This increases our confidence in the ability of the models to successfully identify mountain nyala habitat and we regard them as valuable tools that can contribute to the formulation of conservation and management policies. The suitable areas identified by the model ensemble (Figure 5) are found mostly in regions with montane forest and grassland, situated at relatively high elevation in the Arsi and Ahmar Mountains. This finding agrees with our field observations and the known distributions reported in the literature (Evangelista et al., 2007, 2008, 2015). Based on the findings, the current distributions point to the preferred habitat for mountain nyala being at sites within the montane forest habitat type. According to our ensemble model, the mountain nyala's suitable habitat in the Arsi and Ahmar Mountains was predicted to be approximately 1864 km^2^ (Figure 5). It remains to be established to what extent this estimate corresponds to previous assessments of the species' total distribution range, which has variously been estimated at 5200 (Brown, 1969) and 5087 km^2^ (Payne & Bro‐Jørgensen, 2016b) in the Ethiopian Highlands. While we assume that our models reflect the suitable habitat in the study site, we encourage future validation through field observations to improve the accuracy of the predicted distributions.

Our research analyzed the influence of various environmental factors on habitat suitability for the mountain nyala using both BRT and Maxent models to derive response curves. Land cover type, topographic (e.g., elevation, slope), and vegetation productivity covariates all played distinct roles in determining habitat suitability, but land cover type contributed more than the variables describing topography and vegetation productivity. However, we point out that because the topographic factors can alter microclimatic conditions, they may influence the distribution of land cover types (Obunga et al., 2022) and may thereby also indirectly affect mountain nyala distribution.

Vegetation cover is well‐known to strongly influence the distribution of wild herbivores (Muposhi et al., 2016) and according to both the BRT and Maxent models, land cover type is the most influential environmental variable for the habitat suitability of the species (Table 2). Our findings agree with the notion that mountain nyala prefer forest areas in the south‐eastern highlands of Ethiopia (Brown, 1969) in that the montane forest, vital for provision of cover (Evangelista et al., 2007; Tadesse & Kotler, 2013), was identified as a useful predictor of mountain nyala habitat suitability. So too was grasslands, which provide crucial high‐quality forage. Habitat loss is a primary conservation threat for antelopes across Africa and the effect is exacerbated by climate change, which is estimated to more than half the habitat available for nearly a third of all antelope species by 2080 (Payne & Bro‐Jørgensen, 2016a). Deforestation has resulted in a total loss of 2821 km^*2*^ forest cover in Ethiopia from 2000 to 2012 (Hansen et al., 2013). Deforestation and agricultural expansion have been carried out across a wide span of topographic variation, from gently sloping areas at lower altitude to very steep areas at higher altitude areas (Kindu et al., 2013), and unsustainable forest utilization and poor land use management such as farming of steep areas are aggravating the current situation (Yahya et al., 2020). Hence, it is critical to protect and conserve the remaining forests to protect wildlife in the region (Bishaw, 2001).

Aside from land cover type, NDVI was another key determinant of habitat suitability for the mountain nyala. NDVI provides information on vegetation distribution and is known to often be a strong predictor of animal presence (Pettorelli, 2013). For the mountain nyala, we found higher habitat suitability in for areas with high NDVI (values close to 1). The high NDVI values are associated with dense vegetation, while the low NDVI values are generally associated with non‐continuous vegetation cover (van Bommel et al., 2006). The mountain nyala indeed prefers forested areas with dense vegetation in the Arsi and Ahmar Mountains and high. NDVI may be a proxy for food availability and low visibility for predators (Bradley et al., 2012).

Elevation may also exert an indirect influence on habitat suitability through its impact on landscape diversity and soil dynamics. These factors, in turn, influence vegetation diversity and coverage, which directly affect the suitability of habitat for mountain nyala (Table 2), similar to what has been found in the Bale Mountains (Atickem et al., 2011). As the elevation increases, the relative habitat suitability also increases (Figures 6 and 7) as it directly affects the climatic conditions of a given site (Franklin, 2010). This may be at least partly due to the expansion of agriculture and human settlements at the lower elevations of the Arsi and Ahmar mountains (Evangelista et al., 2007), causing the mountain nyala to move to higher elevations.

Another predictor of mountain nyala habitat suitability in the Arsi and Ahmar Mountains is the slope of the terrain. The relationship between the terrain slope and the habitat suitability for mountain nyala is generally positive (Figures 6 and 7). The preference of mountain nyala for steep slopes might be explained by steep slopes being inaccessible to grazers, whereas the lower flat steppes are subjected to higher anthropogenic disturbance linked to livestock grazing. As a result, mountain nyala may have to seek higher slopes with less disturbance. In a separate study, Walia ibex (*Capra walie*) exploit the rugged topography or steep slopes that allow the species to use at higher altitudes (Gebremedhin et al., 2021).

Despite the limitations, our models still managed to successfully identify several key ecological predictors of mountain nyala habitat suitability; however, the scope of our research was affected by the limited data availability in the country, especially the poor access to spatial data. In particular, we believe that model performances could be improved further if factors such as livestock grazing and human disturbances were integrated into the analyses. A negative correlation between wild ungulates and livestock grazing has been found in many studies (Atickem & Loe, 2014; Bhattacharya & Sathyakumar, 2011; Worku et al., 2023), and livestock grazing is an increasing conservation threat in the Arsi and Ahmar Mountains (Worku et al., 2023). Thus, previous studies have indeed documented that human settlements affect the habitat suitability and behavioral activities of mountain nyala (Atickem et al., 2011; Worku et al., 2021). The effect of human disturbances on ungulate distribution occurs across multiple scales (Schuette et al., 2016), and looking ahead, future research could benefit from incorporating factors such as the extent of human settlements, livestock grazing, and anticipated changes in land use cover of the region into the modeling process.

Integrating the findings of this research with existing conservation frameworks, such as those by the IUCN and regional conservation bodies, can enhance efforts to conserve mountain nyala. The identification of key habitat areas, particularly montane forests and grasslands at higher elevations, should guide effective conservation efforts. Protecting these habitats and mitigating factors like deforestation and agricultural expansion is vital. By understanding which habitats are most suitable for the mountain nyala, strategies can be tailored to protect and restore these areas, including the enhancement of existing protected areas by regulating the intensifying human activities in order to reduce habitat fragmentation and disturbance, key threats to the species' survival.

In conclusion, this study presents the application of two widely used habitat suitability models and contributes significantly to our understanding of the habitat requirements of mountain nyala. Whereas there were slight differences between the two modeling approaches in the habitat suitability maps generates, the ensemble model provides a robust prediction of suitable habitat for the species. More specifically, (1) suitable mountain nyala habitat within the southeastern highlands makes up a small portion of the landscape, accounting for only 9.1% of the study area and (2) land cover type, NDVI, elevation, and slope had the largest influence on habitat suitability of mountain nyala in the Arsi and Ahmar Mountains, Ethiopia. The habitat suitability maps of this study may facilitate the estimation of the total population size in the region and provide important information to guide conservation activities. The study emphasizes the need to protect forested areas and establishes more effective measures to prevent anthropogenic disturbances in order to ensure conservation of wildlife habitats in the Arsi and Ahmar Mountains, Ethiopia.

## AUTHOR CONTRIBUTIONS

**Ejigu Alemayehu Worku:** Conceptualization (lead); data curation (lead); formal analysis (lead); funding acquisition (lead); investigation (lead); methodology (lead); project administration (lead); writing – original draft (lead); writing – review and editing (lead). **Paul H. Evangelista:** Conceptualization (lead); data curation (equal); formal analysis (equal); funding acquisition (equal); methodology (equal); supervision (equal); writing – original draft (equal); writing – review and editing (equal). **Anagaw Atickem:** Conceptualization (lead); data curation (equal); formal analysis (equal); funding acquisition (equal); methodology (equal); supervision (equal); writing – original draft (equal); writing – review and editing (equal). **Afework Bekele:** Conceptualization (equal); data curation (equal); formal analysis (equal); funding acquisition (equal); methodology (equal); supervision (equal); writing – original draft (equal); writing – review and editing (equal). **Jakob Bro‐Jørgensen:** Conceptualization (lead); data curation (equal); formal analysis (equal); funding acquisition (equal); methodology (equal); supervision (equal); writing – original draft (equal); writing – review and editing (equal). **Nils Chr. Stenseth:** Conceptualization (lead); data curation (equal); formal analysis (equal); funding acquisition (equal); methodology (equal); supervision (lead); writing – original draft (equal); writing – review and editing (equal).

## FUNDING INFORMATION

This work was supported by the Rufford Small Grant for Nature Conservation (RSG reference 15774‐1) and IDEA WILD kindly donated field equipment.

## CONFLICT OF INTEREST STATEMENT

The authors declare that they have no known competing financial interests or personal relationships that could have appeared to influence the work reported in this paper.

## Supporting information


Data S1

Table S2

## Data Availability

All data used in the analyses described in this study can be found in the Supporting Information and in the corresponding author.
