# Supplementary material for: Modeling habitat suitability for the lesser‐known populations of endangered mountain nyala (Tragelaphus buxtoni) in the Arsi and Ahmar Mountains, Ethiopia
Source: Ecol Evol. 2024 Apr 15;14(4):e11235. doi: 10.1002/ece3.11235 (PMC11017409; doi:10.1002/ece3.11235)
Supplement: Supplementary file 2 — Table S2 [file ECE3-14-e11235-s001.docx]

**Supplementary Table 2:** Multicollinearity test by using correlation analysis among environmental variables considered for mountain nyala habitat suitability mapping in Arsi and Ahmar Mountains, Ethiopia.

|  | NDVI | Greenness | Wetness | Brightness | Elevation | Slope | Land cover |
| --- | --- | --- | --- | --- | --- | --- | --- |
| NDVI | **1** |  |  |  |  |  |  |
| Greenness | 0.50 | **1** |  |  |  |  |  |
| Wetness | 0.11 | -0.23 | **1** |  |  |  |  |
| **Brightness** | 0.26 | **0.86** | -0.57 | **1** |  |  |  |
| Elevation | -0-16 | 0.04 | -0-59 | 0.27 | **1** |  |  |
| Slope | -0.23 | -0.29 | 0.19 | -0.29 | -0.21 | **1** |  |
| Land cover | 0.15 | 0.26 | 0.27 | 0.19 | -0.57 | -0.1 | **1** |
